# Plasmonic electro-optic modulators on lead zirconate titanate platform

**DOI:** 10.1515/nanoph-2024-0039

**Published:** 2024-03-22

**Authors:** Torgom Yezekyan, Martin Thomaschewski, Paul Conrad Vaagen Thrane, Sergey I. Bozhevolnyi

**Affiliations:** Center for Nano Optics, 6174 University of Southern Denmark, Odense, Denmark; Department of Applied Physics and Materials Science, 6469 California Institute of Technology, Pasadena, USA; Department of Microsystems and Nanotechnology, 509086 SINTEF Oslo, Oslo, Norway

**Keywords:** electro-optic modulators, PZT, plasmonic modulators

## Abstract

The advancement in material platforms exhibiting strong and robust electro-optic effects is crucial for further progress in developing highly efficient and miniaturized optoelectronic components with low power consumption for modern optical communication systems. In this work, we investigate thin-film lead zirconate titanate (PZT) substrates grown by a chemical solution deposition technique as a potential platform for on-chip plasmonic electro-optic modulators. A high modulation depth (>40 %) is achieved with 15 μm-long electro-optic directional coupler modulators. An unusual cutoff in the modulation frequency response at ∼200 kHz is observed and further studied with respect to possible reorientation effects. Second-harmonic generation signals are found influenced by the externally applied electric field, indicating that the domain reorientation effect can be responsible for the unusual frequency response observed.

## 1 Introduction

Given ever going increase in data traffic and advancements in optical interconnect technology, there is a persistent demand for developing electro-optic modulators that are both energy-efficient and capable of high-speed operation with minimal losses. In pursuit of this objective, various electro-optic phenomena are harnessed, including the plasma dispersion effect [1], the quantum-confined Stark effect [2], and the linear electro-optic (Pockels) effect [3]. The Pockels effect is most used for optical modulation in telecommunication technology due to its practically instantaneous response, enabling high speed operation, and linear response with respect to the applied control electric field. Therefore, it is crucially important to explore electro-optic material platforms that are characterized by high environmental stability, facile fabrication process, seamless integration capabilities, and high efficiency. Presently, the predominant Pockels material employed in electro-optic modulators is lithium niobate (LN) [4], which features, however, a medium electro-optic coefficient (*r*_33_ = 31 pm/V), resulting typically in millimeter-long integrated optical modulators, a circumstance that makes the design of high-speed modulators challenging [5]. One of the ways to reduce the interaction length and thereby increase the modulation speed (bandwidth) is to exploit materials with stronger electro-optic effects, i.e., with higher Pockels coefficients. This approach has led to intensive research in somewhat exotic materials, such as electro-optic polymers, for which electro-optic coefficients were found to exceed those of LN by a factor of 10 or higher [6], [7] or thin-film barium titanate exhibiting one of the largest electro-optic coefficients (∼1000 pm/V) [8], [9]. However, achieving uniformity and reproducibility in large-scale production of electro-optic polymers poses a significant challenge. In addition, these materials are strongly affected by the ambient factors, such as temperature and humidity, making their long-term performance unstable. Further concerns about barium titanate thin films include complex and expensive bonding fabrication and high propagation loss (∼10 dB/cm) [10]. Hence, there is still an ongoing search for novel material platforms that would ensure highly efficient and robust electro-optic modulation.

Lead zirconate titanate (PZT) films have been widely used as transducers in electronics because of their physical strength, high operating temperature, chemical inertness, and their relatively low manufacturing cost. PZT films were also reported to exhibit high Pockels coefficients (∼220 pm/V [11], ∼150 pm/V [12], and ∼133 pm/V [13]), which are considerably larger than those of LN. Since PZT substrates are relatively inexpensive and can easily be mass-produced, they seem to be very interesting candidates for realizing electro-optic modulators with significantly improved characteristics. It is worth to mention, though, that the substrate composition and the actual technique of the PZT fabrication may strongly affect the overall operation of the modulators [11].

In addition to the material platform, another way of boosting the modulator performance (efficiency and bandwidth) is to make use of unique properties of plasmonic modulators. Employing the same metallic circuitry for both guiding surface plasmon–polariton (SPP) modes and delivering control electrical signals guarantees a significant overlap of the SPP mode field and the locally induced (by control signals) change in the refractive index [14]. The inherent feature of seamless electric–photonic interfacing in SPP-based electrically controlled components has paved the way for highly efficient plasmonic electro-optic modulators [14], [15], [16]. Given a suitable (efficient and robust) material platform, plasmonics brings unprecedented potentials for providing efficient, ultracompact, and ultrafast electro-optic modulators and switches.

**Figure 1:**
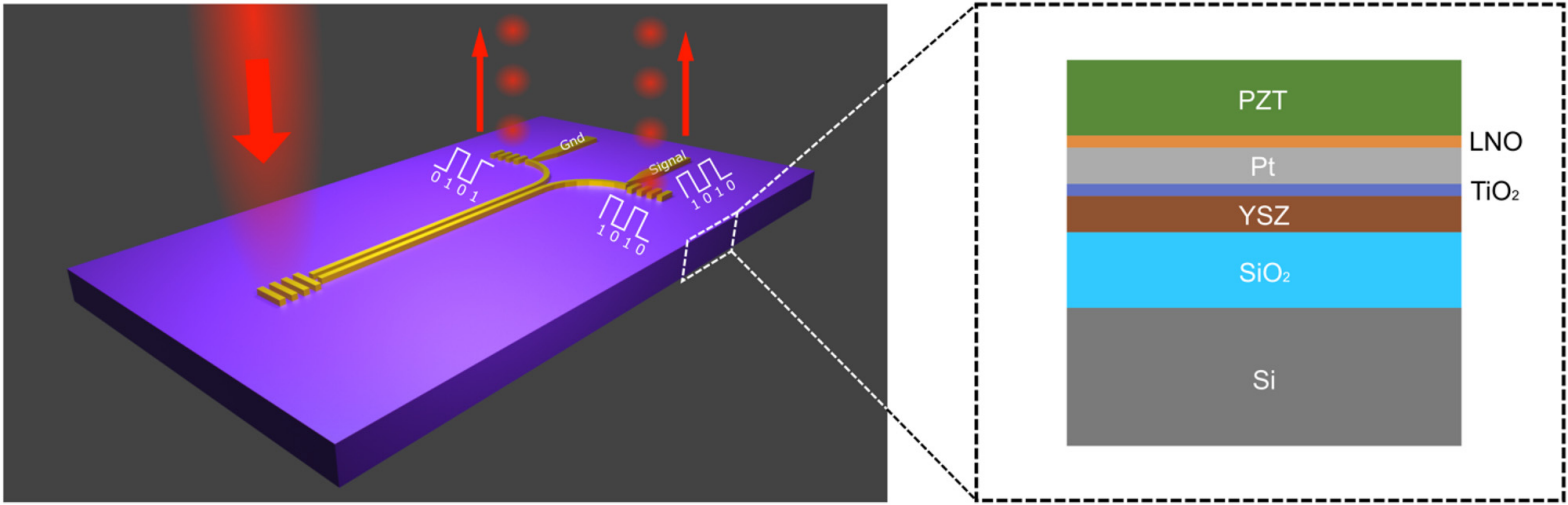
Schematic of a plasmonic PZT directional coupler modulator consisting of electrically connected gold stripes, supporting the SPP propagation and terminated with in- and out-coupling gratings (a). Layered structure of the PZT wafer (b); layer thicknesses are shown not to scale.

In this work, we investigate monolithic plasmonic electro-optic modulators realized with the thin-film PZT platform, which is widely used for piezoelectric applications. To test the modulator performance, we used the “gold standard” LN as a reference; hence, the design of modulators on the PZT platform is chosen to be identical to the plasmonic directional coupler switches realized previously with the LN platform [16]. The modulator configuration is based on two identical gold nano-stripes fabricated atop PZT, with the stripes acting as both SPP waveguides, forming a directional coupler, and metal electrodes for applying external electric fields (Figure 1(a)). In this configuration, the anticipated antisymmetric change of substrate refractive index, due to the electro-optic effect induced by an external electric field applied across two gold nano-stripes, affects differently the two global SPP stripe (odd and even) modes propagating along the two-stripe SPP waveguide [16]. Their interference influenced by applied electrical signals results in electrically controlled switching of the SPP waveguide power delivered to two out-couplers, with equal distribution of the output powers when no electrical signals are applied. This compact configuration enables high-density integration of plasmonic directional couplers, facilitating efficient and broadband switching of optical power distribution at telecom wavelengths between the two output ports.

## 2 PZT substrate fabrication

The PZT deposition process that was used has previously been optimized for MEMS applications, where the PZT thin film is sandwiched between a lower and upper electrode, Pt and Au, respectively, with the voltage being applied across the film. In the current application, the voltage is applied between two electrodes that are on the same (upper) side of the PZT thin film. In the configuration studied in this work, the bottom Pt layer is undesirable because it leads to an additional capacitance that may limit the upper modulation frequency. However, this Pt layer together with a seed layer of lanthanum nickel oxide (LNO) is used to promote the desired (001) orientation of the PZT polycrystalline structure [17]. To address both these considerations, the bottom Pt layer was patterned in such a manner that it was only present underneath the areas, where the waveguide electrodes are present and there is a need for correctly oriented PZT, while the Pt layer was not present underneath the macroscopic electrodes used for contacting the SPP circuitry (otherwise these would stand for most of the capacitance). A layer of yttria-stabilized zirconia (YSZ) was added atop a thick SiO_2_ layer, functioning as a lattice matching layer to allow the PZT to be deposited also in areas where the Pt layer was absent (Figure 1(b)). To further reduce capacitance associated with the macroscopic top electrodes, a thick layer of SiO_2_ is added to increase the separation of these electrodes from the high resistive Si wafer that was used. Unfortunately, silica glass (SiO_2_) wafers could not be used due to high temperatures used during Ti oxidation and PZT deposition. Fused quartz and sapphire wafers were considered but were not readily available at the time of fabrication, which is a potential point of improvement. Different layers of the fabricated substrate are shown in Figure 1(b), including an adhesion layer of TiO_2_ underneath the Pt electrode.

The fabrication steps are as follows: high resistive Si wafers (Topsil, 150 mm diameter, 15,000–30,000 Ω/cm) were thermally oxidized giving a SiO_2_ thickness of 2.4 µm, followed by the deposition of 100 nm of YSZ using pulsed laser deposition. Next, a thin layer of Ti was deposited by metal sputtering and subsequently thermally oxidized to TiO_2_. Then, a 100 nm thick Pt layer was sputtered onto the wafer, together with a seed layer of LNO applied using chemical solution deposition. This bottom electrode was patterned using photolithography and etching in HCl (to remove LNO) and reactive ion etching (to remove Pt). Following this, 2 µm of PZT was put down using chemical solution deposition (SolarSemi CSD-PZT tool, with MMC PZT-Nb solution). Note that this describes the fabrication of the second round of PZT substrates as described below, while the first round of substrates was different in that there was no YSZ, no patterning of Pt/LNO, and the PZT thickness was 800 nm.

## 3 Plasmonic modulator

**Figure 2:**
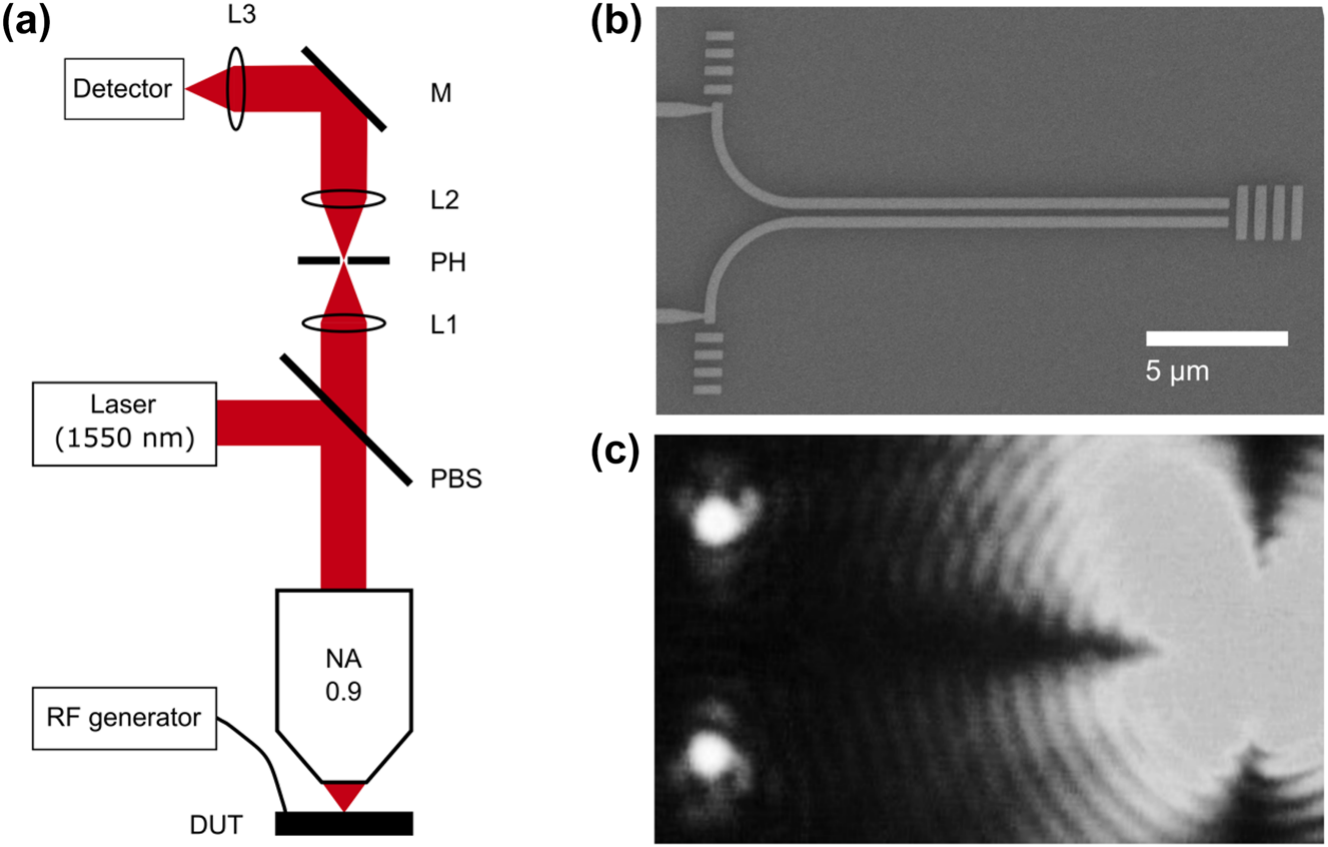
Schematics of the setup used for modulation measurements (a). SEM image of an individual 15-µm-long plasmonic modulator, consisting of two identical parallel 350-nm-wide gold stripes separated by a gap of 300 nm (b). Optical far-field image of the unbiased modulator illuminated with a diffraction limited spot (wavelength of 1550 nm) positioned at the in-coupling grating (c). The scale bar in (c) is the same as in (b).

To test the electro-optic modulation properties of the fabricated PZT substrates, plasmonic modulators were fabricated on them, closely following the device parameters, implemented when developing plasmonic directional coupler switches realized on a LN platform [16]. The design concept is based on using two identical metal (e.g., gold) stripes fabricated atop an electro-optic material (LN or PZT) with the optical axis normal to the surface so that the same metal structure can be used for both supporting the SPP propagation and controlling the interference of guided SPP modes with externally applied electrostatic fields (utilizing the strongest electro-optic effect). An exceptionally simple device architecture (Figure 1(a)) makes this design very interesting and appealing for high-density integration of electrically controlled nanophotonic components. When no voltage is applied, the symmetric coupler configuration ensures that the same power is delivered to the two out-couplers of the modulator. However, when a voltage is applied, the propagation constants associated with the two global SPP stripe modes are differently changed influencing their interference at the output, and one can achieve a complete (for certain coupler lengths) switch of power between two out-couplers [16]. The designed configuration involved two parallel 50-nm-thick and 350-nm-wide gold stripes separated by a gap of 300 nm with the overall length of 15 µm. The plasmonic PZT modulators were characterized in the telecom wavelength range using the optical setup assembled for this purpose (Figure 2(a)). A polarized collimated telecom laser beam (wavelength of 1550 nm) is passing through a polarizing beam splitter and IR-objective (×100 magnification, NA = 0.95). The device under test (DUT) is attached to a 3D piezo stage for precise positioning. The electric signal is carried to the DUT by a RF probe with ground-signal-ground configuration (GGB, Model 40A-GSG-750, working range: DC – 40 GHz).

Fabrication of the device relies on a two-step lithographic process using the mix-and-match technique for lithographic overlay. First, connecting electrodes [microwave (RF) coplanar waveguides] are patterned onto a PZT chip by photolithography, metal deposition (5 nm Ti + 100 nm Au), and lift-off. Subsequently, the stripe circuitry of modulators is configured using the electron-beam writing at an acceleration voltage of 30 keV in 200-nm-thick PMMA positive resist and 60-nm-thick conductive polymer (AR-PC 5090.02) to dissipate charge accumulation on the sample surface. After the resist development, the modulator circuitry is formed by depositing a 2-nm-thin titanium adhesion layer and a 50-nm-thick gold layer by thermal evaporation, followed by subsequent 12 h lift-off in acetone. The alignment between the two lithography steps is performed manually using the corners of the coplanar waveguide stripes. The overall quality of the modulator electrode structure along with in- and out-coupling gratings is judged upon using SEM images (Figure 2(b)).

During the optical characterization, the DUT was precisely positioned so that the incident focused laser beam polarized along the propagation direction (this polarization ensures the most efficient SPP excitation [16]) illuminated symmetrically the input grating coupler. In this case, the power at the output grating couplers was equally distributed (Figure 2(c)). Due to the 90°-bends of the plasmonic waveguides, the polarization of the outcoupled radiation was orthogonal to that of the incident beam, a configuration that allowed us to suppress the back-reflection of the incident beam in the detection part (e.g., by a polarizer or a polarizing beam splitter). The well-pronounced radiation output spots seen on the optical image (Figure 2(c)) were very similar in appearance and contrast to those observed with the LN plasmonic modulator [16], indicating a similar level of optical performance (including the insertion losses). To estimate the propagation length of the SPP mode, four individual plasmonic waveguides were fabricated with different lengths (10 μm, 15 μm, 20 μm, and 25 μm, respectively) and identical in/out-couplers; consequently, the transmitted power through the waveguides was measured. The measured propagation length of the SPP waveguide is 12 μm, which is around 2.5 times larger than the one obtained for LN [16]. Given the length of the plasmonic waveguide of 15 µm, the insertion loss due to the propagation losses is 5.4 dB. We further concentrated our measurements on the frequency response characterization of the PZT plasmonic modulators. For that purpose, the optical signal from one of the arms of the plasmonic modulator was further spatially filtered at the image plane (after the lens L1) with a pinhole PH and directed to an InGaAs high-speed amplified photodetector (Figure 2(a)). The frequency response, i.e., the modulation depth as a function of the modulation frequency, was measured with an oscilloscope (bandwidth of 200 MHz).

**Figure 3:**
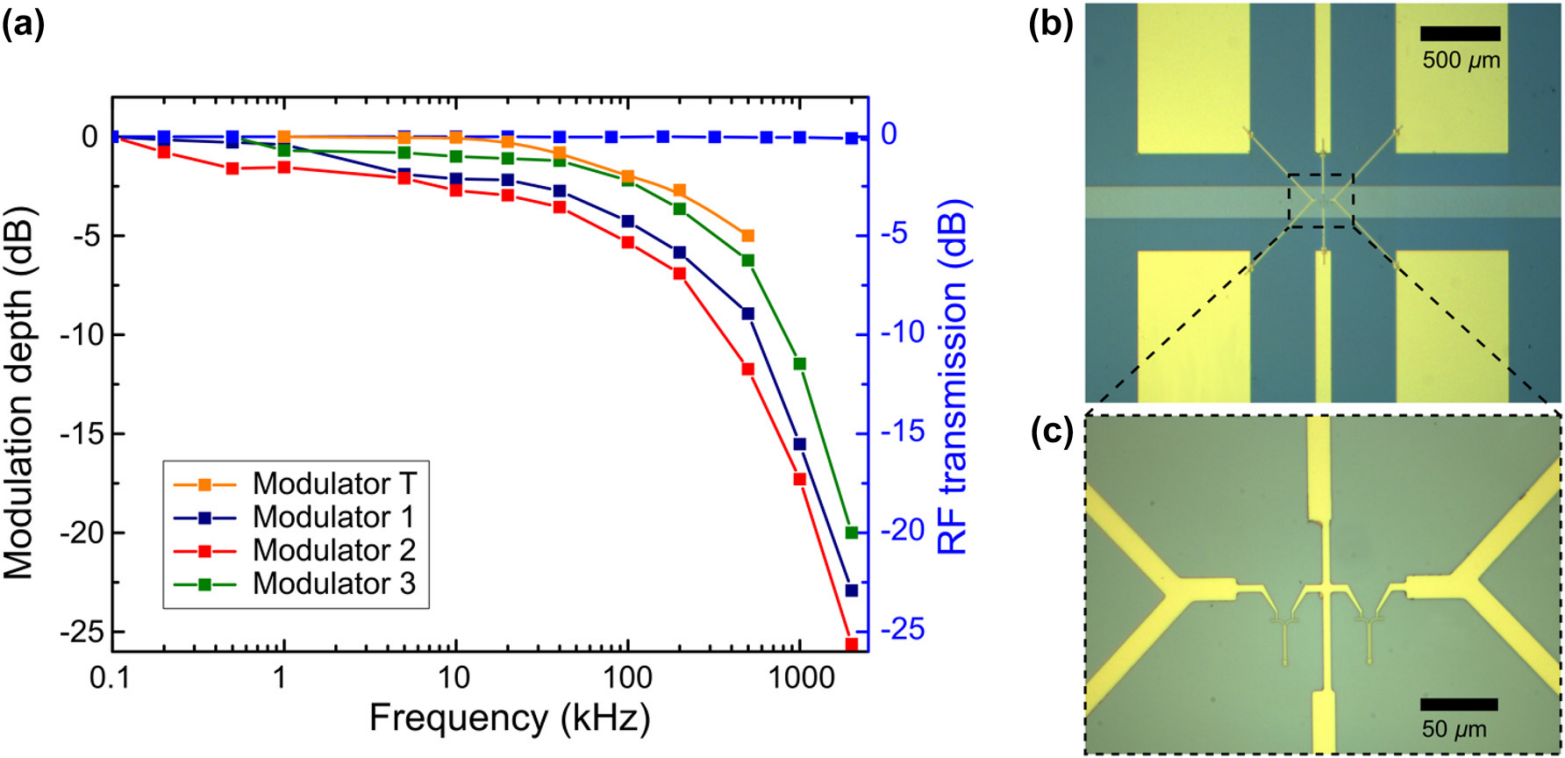
Frequency responses of several devices along with the RF transmission of a device (blue symbols), including modulators and RF waveguides (a). Microscope images of the device used for the RF transmission measurements emphasizing the symmetrical RF waveguides (b) and plasmonic modulators connected in between (c).

For the first round of measurements, plasmonic modulators were fabricated on a substrate with Pt and LNO layers covering the whole area. Although a high modulation depth of 23 % was obtained with the driving voltage of 15 V (the modulation depth obtained for the same driving voltage on LN was ∼14 % [16]), the modulator frequency response exhibited a very low cutoff at around 200 kHz (see orange symbols for modulator T in Figure 3(a)). This behavior was suspected to be a consequence of the device capacity, since the PZT dielectric constant is very large. To clarify this issue, the capacitance of the device was estimated by treating the metal layer (Pt) beneath the PZT film as an intermediate plate of a capacitor, with the signal and ground electrodes being terminals (a model of two capacitors connected in series). With the dielectric permittivity of PZT equal to 2000 [17], the PZT thickness of 800 nm, and the area of electrodes being approximately 6 mm^2^, the estimated capacitance is 16 nF, leading to −3 dB frequency limit of approximately 200 kHz at *R* = 50 Ω resistive load. However, it is worth mentioning that variations in the reported values of PZT dielectric permittivity are very large: from 500 to 12,000 [17], [18], [19].

To overcome the limitations caused by the possible capacitive effects, a new PZT wafer was fabricated, where the Pt and LNO layers were removed, except for small areas, and the plasmonic modulators were fabricated, appearing as a slightly deem (as compared to top gold electrodes) horizontal stripe in optical images (Figure 3(b)). This means that there was no more overlap between the large electrodes (RF waveguide) and the metal layer underneath. Three different samples were prepared, with plasmonic modulators fabricated on the new substrates and similarly large modulation depths were achieved (24 %, 35 %, and 41 %). However, the same frequency limitation at around 200 kHz was observed (modulators 1, 2, and 3 in Figure 3(a)).

To test the possible occurrence of low-pass filtration in the RF part of the sample, namely, from the electrodes to the modulators, two identical RF waveguides were fabricated (Figure 3(b)), and the modulator was connected to both RF waveguides (Figure 3(c)). This imitates a RF transmission line with a capacitor connected between the signal and ground line, where the capacitor is formed by the plasmonic modulator. Two identical probes were attached to the two RF waveguides from each side, and the electrical signal transmission was measured, revealing lossless RF transmission up to >2 MHz (Figure 3(a)). Since the electro-optic Pockels effect is inherently fast, we were forced to consider other mechanisms involved in the observed electric modulation of optical signals.

Besides the Pockels effect, one can think of two other contributions that may lead to an electro-optic effect: the elasto-optic effect (mechanical deformation of the unit cell), and a contribution arising from the change in the polarization direction due to the ferroelectric domain switching caused by domain wall motion. Both effects are relatively slow and would be cut off at high frequencies. These effects are also rather strong and might cause the observed modulation, at least partially contributing to the overall effect observed, and therefore should further be investigated.

Apart from the low cut-off frequency one should note a large variation between the modulation depths for different devices (i.e., nearly two-fold difference between modulator 1 and modulator 3). On the other hand, this might be a result of non-homogeneity in the PZT substrate, namely, the small sizes and random distribution of domains.

## 4 Domain characterization

It is well known that the crystal phases and substrates, on which the PZT film is grown, may highly influence the optical and electro-optical properties of the latter [11]. The high piezoelectricity of PZT originates in the existence of a monoclinic crystal structure and the formation of microscopic domains at the morphotropic phase boundary [20]. The size and orientation of the domains within the PZT layer is an important characteristic since it determines the overall performance of modulators. As described in the preceding section, during the growth process, several layers on the substrate were specifically implemented to increase the domain size of the PZT film (with lateral sizes as large as several micrometers). Furthermore, the resulted film was expected to have a primarily out of plane-oriented domains.

The typical method to evaluate domain structures of ferroelectrics is transmission electron microscopy (TEM), which provides high spatial resolution to visualize the local domain structures [21]. However, the TEM images would still have to be properly treated for accurately determining domain volume fractions, especially for the in-plane domains in the disordered multi-domain system. Furthermore, the TEM is a sophisticated characterization technique with a complicate process of specimen preparation, which usually presents the domain structures at a local region far from the whole picture of the domains in the sample. On the other hand, the second harmonic generation (SHG) microscopy is a promising approach for locally probing domain structures in ferroelectrics at places of interest, as the SHG response is highly sensitive to the polarization angle of the incident light with respect to ferroelectric domain orientations [22], [23].

**Figure 4:**
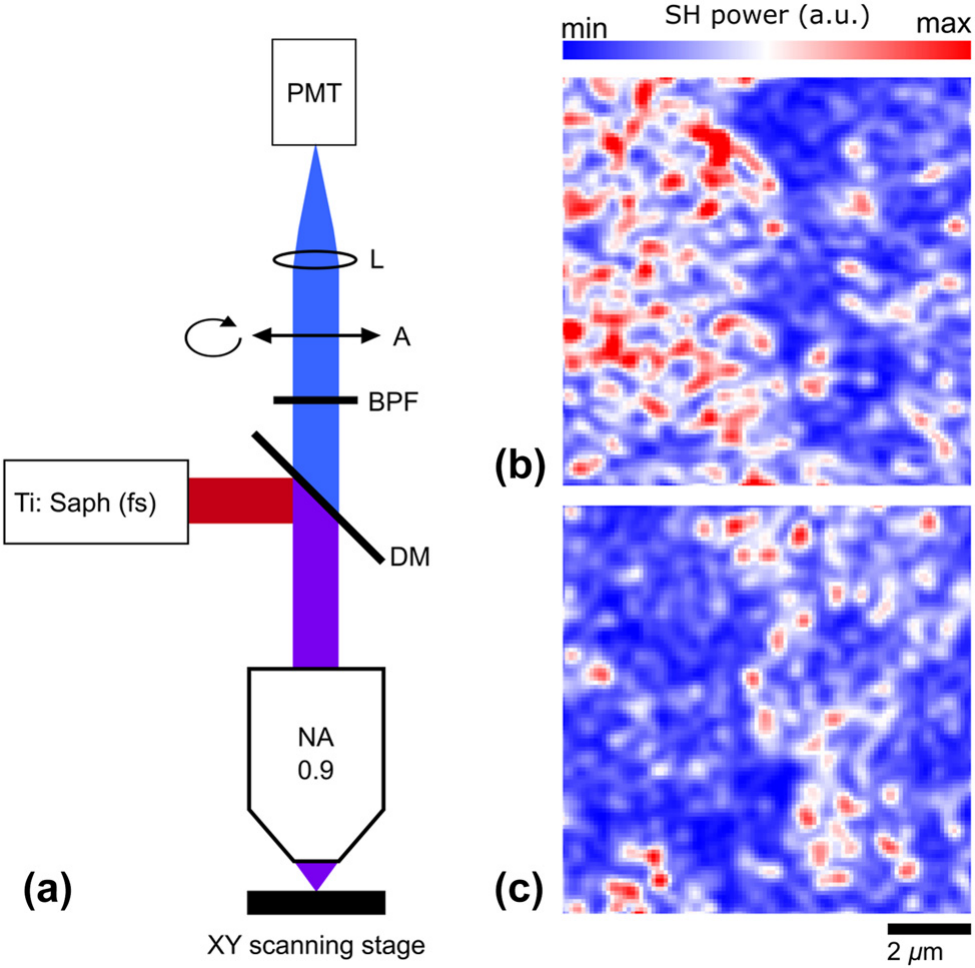
Schematics of the SH generation measurement setup (a). SH intensity scans of the same PZT region with an analyzer aligned parallel (b) and perpendicular (c) to the polarization of the incident laser beam.

To investigate the properties of thin-film PZT, we implemented a homebuilt scanning nonlinear microscope (Figure 4(a)), enabling investigations of local polarization characteristics of the SHG radiation. A linearly polarized pulsed laser beam (tunable wavelength set at 800 nm and pulse duration of ∼100 fs) was focused on the sample with an apochromatic objective (NA = 0.9). The nonlinear reflection was collected with the same objective and filtered out using a dichroic mirror (DM) and a band-pass filter (BPF) at 400 nm. The polarization of the SH was resolved using an analyzer (A), whereas the SH signal was recorded using a photo-multiplier tube.

The SH images of the same area of the PZT film, with the analyzer being aligned parallel or perpendicular to the polarization of the incident laser beam, were recorded (Figure 4(b) and (c), respectively). One notices that the two maps are somewhat complimentary indicating, contrary to the expectations from the growth process, the existence of in-plane oriented domains, with different in-plane orientations (on both maps, the maximum SH power is one order of magnitude larger than the minimum measured power). The domain sizes in our samples were estimated to be rather small, close to the resolution limit of ∼500 nm our setup, which means that the overall efficiency of the modulator would be largely reduced since a single domain device fabrication could not be realized. Another drawback is related to the circumstance that the design of the proposed plasmonic modulator requires a unidirectional out-of-plane domain orientation for achieving the best performance [16], which seem not to be fully realized as judged from the SH experiments (Figure 4(b) and (c)). We observe clear indications of multi-domain ferroelectric responses for the in-plane components of the investigated PZT thin films. This suggests that the out-of-plane non-linear coefficients of the ferroelectric domains have an equally non-collective orientation over distances much larger than the domain size, resulting in a varying electro-optic response depending on the specific location on the substrate. These circumstances are thought to be main factors resulting in the performance alteration from one device to another.

## 5 Domain reorientation

**Figure 5:**
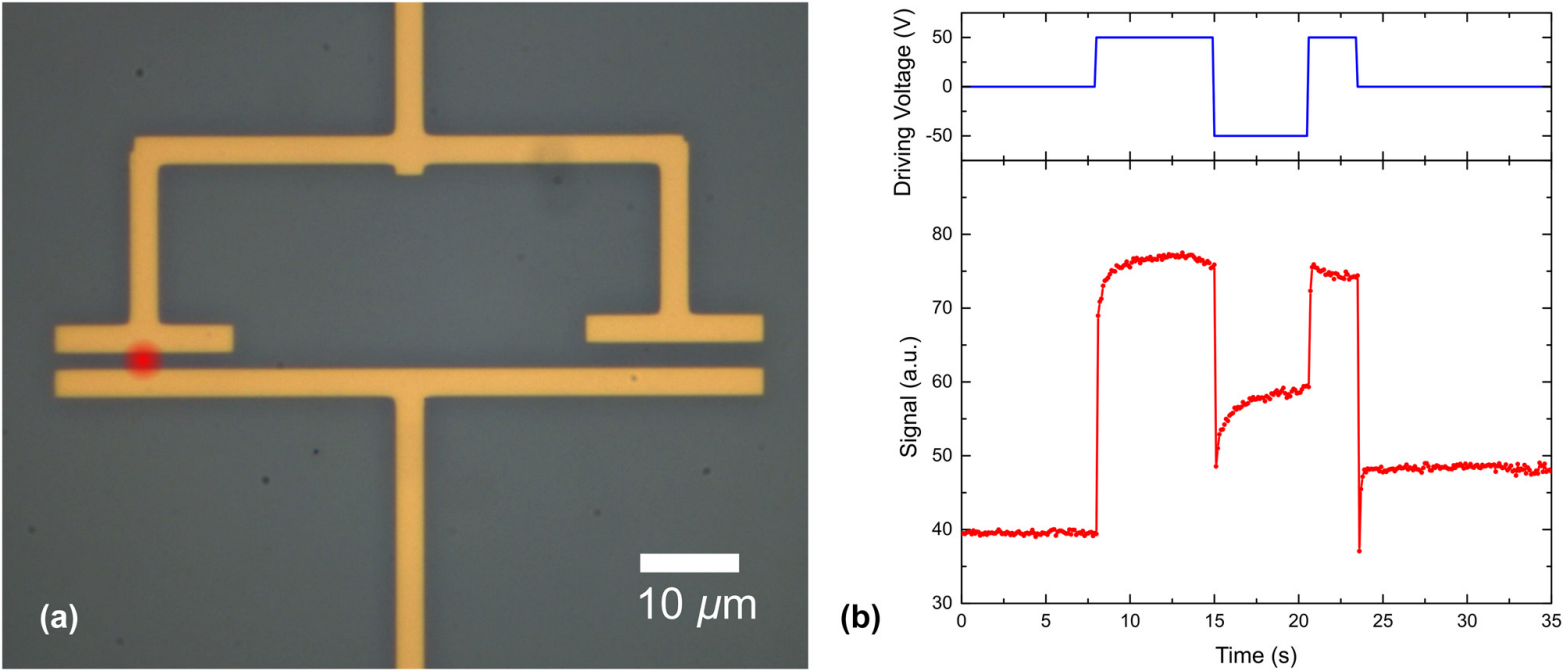
Microscope image of the gold electrode structure used for the PZT poling test (a). The red spot denotes the position of the incident FH laser beam polarized across the electrode gap, i.e., parallel to the applied electric field. The SH response to the externally applied driving (poling) voltage (b). The SH power was measured in reflection using the setup shown in Figure 4(a).

In the optical characterization experiments described above, the plasmonic modulators were tested at the driving voltage of 15 V, a value that, given the SPP guiding stripe separation of 300 nm, implies generation of strong electrostatic fields of ∼500 kV/cm inside the material. This value is quite large and might cause certain poling effects in the PZT layer (for example, PZT was reported to be poled at 150 kV/cm [24]). To verify the occurrence of the domain reorientation effect stimulated by an external electric field and thereby better understand physical mechanisms involved in the observed modulation, we have fabricated a dedicated electrode structure atop the thin-film PZT (Figure 5(a)). The structure consists of two gold stripes (electrodes) arranged to form two capacitors with gaps of 1 and 3 µm, which are connected in parallel.

The setup used for the poling test was essentially the same as that used for the SH generation measurements revealing inhomogeneity in the PZT domain orientation (Figure 4), apart from a different objective (×20 objective, NA = 0.4) and removed analyzer. A focused FH laser beam was positioned between the gold electrodes, being polarized in the direction perpendicular to the electrodes, i.e., parallel to the externally applied electric field. The evolution of SH power was monitored when positive and negative bias voltages were applied to the electrodes (Figure 5(b)). To generate electro-static fields in the PZT similar to those created in plasmonic modulator structures, voltages of ±50 V were applied to the system of electrodes. Due to technical limitations, namely, the speed of the photodetector and the absence of possibility to synchronize the photodetector with a function generator, the switch between different bias voltages was done manually, recording the generated SH power. We have observed many SH power dependencies in time that were all featuring qualitatively similar changes whenever the bias voltage was changed (Figure 5(b)). Quantitatively, these changes were very poorly reproduced with the electrodes being always (thermally) destroyed after a few cycles. Nevertheless, the fact of domain reorientation due to bias voltage changes seen in the associated SH power changes observed with our thin-film PZT films is indisputable. At the same time, we believe that, for different procedures of PZT fabrication, this effect might be strongly suppressed while significantly extending the bandwidth of optical modulation [9].

## 6 Conclusions

We have thoroughly investigated thin-film PZT substrates grown by a chemical solution deposition technique as a potential platform for on-chip plasmonic electro-optic modulators. To characterize the domain orientational distribution and sizes, we implemented scanning nonlinear microscopy operating in reflection. It was found that the fabricated PZT substrates contain microscopic domains exhibiting, despite the predictions from the fabrication process, significant in-plane domain orientation. Nevertheless, 15-μm-long plasmonic directional coupler modulators fabricated on thin-film PZT substrates demonstrated a large modulation depth (>40 %) for the driving voltage of 15 V, a performance exceeding that observed with similar modulators on LN substrates [16]. However, a low frequency cutoff at ∼200 kHz was found even with special PZT substrates fabricated to decrease the capacitance of the electrode configuration. RF transmission of the system with modulators connected in parallel was measured, and no drop in transmission was observed at least up to 2 MHz. Furthermore, a special two-electrode structure (gap ∼1 µm) was fabricated on the same PZT substrate to investigate the domain reorientation effect under an externally applied electric field of ∼500 kV/cm. Evolution of the detected SH power was recorded when the external bias voltage of 50 V was switched off and reversed. We observed significant step-like changes in the detected SH signal whenever the bias voltage was changed, indicating unambiguously the domain switching effect. Considering the circumstance that the crystal phases and substrates, on which the PZT film is grown, influences its electro-optical properties [11], we believe that, with the improved PZT fabrication and processing, unintentional poling effects can be suppressed and the bandwidth and depth of optical modulation can be significantly extended by harvesting the large electro-optic coefficients of PZT [9]. This would open an exciting possibility for realizing ultra-compact, stable, efficient, and fast electro-optic plasmonic components with inexpensive thin-film PZT substrates.

## References

[1] Han J.-H., Boeuf F., Fujikata J., Takahashi S., Takagi S., Takenaka M. (2017). Efficient low-loss InGaAsP/Si hybrid MOS optical modulator. *Nat. Photonics*.

[2] Chaisakul P. (2014). Integrated germanium optical interconnects on silicon substrates. *Nat. Photonics*.

[3] Chen L., Xu Q., Wood M. G., Reano R. M. (2014). Hybrid silicon and lithium niobate electro-optical ring modulator. *Optica*.

[4] Li M., Ling J., He Y., Javid U. A., Xue S., Lin Q. (2020). Lithium niobate photonic-crystal electro-optic modulator. *Nat. Commun.*.

[5] He M. (2019). High-performance hybrid silicon and lithium niobate Mach–Zehnder modulators for 100 Gbits− 1 and beyond. *Nat. Photonics*.

[6] Haffner C. (2017). Harnessing nonlinearities near material absorption resonances for reducing losses in plasmonic modulators. *Opt. Mater. Express*.

[7] Heni W. (2017). Nonlinearities of organic electro-optic materials in nanoscale slots and implications for the optimum modulator design. *Opt. Express*.

[8] Bernasconi P., Zgonik M., Günter P. (1995). Temperature dependence and dispersion of electro-optic and elasto-optic effect in perovskite crystals. *J. Appl. Phys.*.

[9] Winiger J. (2023). PLD epitaxial thin-film BaTiO3 on MgO− dielectric and electro-optic properties. *Adv. Mater. Interfaces*.

[10] Ortmann J. E. (2019). Ultra-low-power tuning in hybrid barium titanate–silicon nitride electro-optic devices on silicon. *ACS Photonics*.

[11] Zhu M., Du Z., Ma J. (2010). Influence of crystal phase and transparent substrates on electro-optic properties of lead zirconate titanate films. *J. Appl. Phys.*.

[12] Choi J.-J., Park G.-T., Kim H.-E. (2004). Electrooptic properties of highly oriented Pb (Zr, Ti) O_3_ film grown on glass substrate using lanthanum nitrate as a buffer layer. *J. Mater. Res.*.

[13] Ban D., Liu G., Yu H., Sun X., Deng N., Qiu F. (2021). High electro-optic coefficient lead zirconate titanate films toward low-power and compact modulators. *Opt. Mater. Express*.

[14] Haffner C. (2015). All-plasmonic Mach–Zehnder modulator enabling optical high-speed communication at the microscale. *Nat. Photonics*.

[15] Melikyan A. (2014). High-speed plasmonic phase modulators. *Nat. Photonics*.

[16] Thomaschewski M., Zenin V. A., Wolff C., Bozhevolnyi S. I. (2020). Plasmonic monolithic lithium niobate directional coupler switches. *Nat. Commun.*.

[17] Basu T., Sen S., Seal A., Sen A. (2016). Temperature dependent electrical properties of PZT wafer. *J. Electron. Mater.*.

[18] Dorey R., Whatmore R. (2004). Electrical properties of high density PZT and PMN–PT/PZT thick films produced using ComFi technology. *J. Eur. Ceram. Soc.*.

[19] Park H.-H., Yoon S., Park H.-H., Hill R. H. (2004). Electrical properties of PZT thin films by photochemical deposition. *Thin Solid Films*.

[20] Everhardt A. S., Matzen S., Domingo N., Catalan G., Noheda B. (2016). Ferroelectric domain structures in low-strain BaTiO_3_. *Adv. Electron. Mater.*.

[21] Ding Y., Liu J., Wang Y. (2000). Transmission electron microscopy study on ferroelectric domain structure in SrBi_2_Ta_2_O_9_ ceramics. *Appl. Phys. Lett.*.

[22] Denev S. A., Lummen T. T., Barnes E., Kumar A., Gopalan V. (2011). Probing ferroelectrics using optical second harmonic generation. *J. Am. Ceram. Soc*..

[23] Zhang Y. (2018). Characterization of domain distributions by second harmonic generation in ferroelectrics. *Npj Comput. Mater.*.

[24] Alexander K. (2018). Nanophotonic Pockels modulators on a silicon nitride platform. *Nat. Commun.*.

